# Next steps in the early detection of ovarian cancer

**DOI:** 10.1038/s43856-021-00037-9

**Published:** 2021-10-05

**Authors:** Robert C. Bast, Chae Young Han, Zhen Lu, Karen H. Lu

**Affiliations:** 1 Department of Experimental Therapeutics, University of Texas M.D. Anderson Cancer Center, Houston, TX 77030 USA; 2 Department of Gynecologic Oncology and Reproductive Medicine, University of Texas M.D. Anderson Cancer Center, Houston, TX 77030 USA

**Keywords:** Ovarian cancer, Diagnostic markers

## Abstract

Bast et al. discuss the early detection of ovarian cancer in the context of the recent UKCTOCS screening trial. The authors suggest potential reasons why the trial failed to achieve a reduction in mortality and outline next steps in the development of biomarkers and imaging modalities to detect ovarian cancer.

## Teaser

A recent ovarian cancer screening trial found no reduction in mortality, despite increased detection of early stage disease. Here, we discuss these findings and examine next steps to develop more effective approaches for the early detection of ovarian cancer.

Ovarian cancer afflicts more than 300,000 women each year worldwide. Despite improved care with cytoreductive surgery and combination chemotherapy, the majority of patients will die from their disease. When cancer is limited to the ovaries in stage I, up to 90% of patients can be cured with currently available treatment^1^. Even when disease has spread to pelvic organs in stage II, up to 70% survive for more than 10 years. With further spread over the surface of the abdominal cavity (stage III) or outside the abdomen (stage IV), long term survival is reduced to 20% or lower. Approximately 25–30% of patients are currently diagnosed in stage I or II. It has long been assumed that increasing the fraction of women with ovarian cancer detected at an early stage could improve survival and decrease mortality.

The negative outcome of the UKCTOCS calls into question where we should go next to find an effective strategy for early detection of ovarian cancer.

## The United Kingdom Collaborative Trial of Ovarian Cancer Screening (UKCTOCS)

The UKCTOCS, the largest ovarian cancer screening trial conducted to date, randomized postmenopausal women at average risk for developing ovarian cancer to a control group (101,314), annual transvaginal sonography (TVS) for 7 years (50,623), or “multi-modal screening” for 7 years (50,625) involving a “two-step process”, where changes in annual CA125 ovarian tumor biomarker blood tests were analyzed with a Bayesian Risk of Ovarian Cancer Algorithm (ROCA) prompting TVS in a small fraction of patients with a significant increase in CA125^2^. Executing a screening study of this magnitude constitutes a remarkable achievement. In the initial report in 2016, there was no significant reduction in mortality overall, but a 20% reduction was found in a pre-specified subset of women with incident disease who had been diagnosed after 7 years of screening on the multi-modal arm (*p* = 0.021). Given wide statistical bounds around the estimate, re-analysis was planned after 5 years of additional follow-up. The update confirmed a stage shift with an increase in early stage disease and a decrease in late stage disease in the screened population, but failed to confirm a reduction in mortality^1^.

Failure to sustain a mortality advantage despite an increase in early stage disease could relate to inadequate therapy. As collaborating sites were chosen for expertize in gynecologic oncology, surgery is likely to have been state-of-the-art. It would be important to know that all early stage patients received six cycles of carboplatin and paclitaxel. If the choice and duration of chemotherapy were at the discretion of collaborating oncologists, some might have chosen single agent carboplatin or used only three cycles of combination chemotherapy for early stage disease.

Early stage (I/II) disease detected in the multimodal arm of the UKCTOCS was associated with increased mortality, consistent with the possibility that rising CA125 detected additional micro-metastatic disease or identified visible tumors that resisted conventional chemotherapy. While PARP-inhibitor maintenance therapy is generally prescribed for advanced stage (III/IV) disease, patients with screen-detected homologous repair deficient early stage (I/II) ovarian cancer might also benefit. Novel agents, including SIK2 inhibitors^3^, are being developed to enhance primary treatment with carboplatin and paclitaxel potentially improving care for both early and late stage disease.

Furthermore, the magnitude of the stage shift observed in the UKCTOCS may not have been sufficient to reduce mortality. The fraction of stage I/II patients in the UKCTOCS increased from 28.4% with no screening to 38.1% with CA125 followed by TVS. The fraction of patients with stage IV disease decreased from 20.7 to 15.1%. A much greater stage shift was observed in the single arm Normal Risk Ovarian Screening Study (NROSS) that has been conducted, in parallel, over the last 19 years in post-menopausal women at average risk in the United States, using the identical two-step multi-modal screening plan with the CA125 based ROCA followed by TVS. Among the 7597 women screened, 16 epithelial ovarian cancers have been detected—2 were borderline and 14 invasive—with 11 (69%) in early stage (I or II) (updated from ref. ^4^). One of 16 cases (6%) was detected in stage IV. Both trials confirmed that adequate specificity could be attained with the two-step strategy, requiring no more than 2–4 operations to detect each case of ovarian cancer. The reason for a greater stage shift in the smaller trial is not clear. This could reflect statistical variation with the smaller size of the NROSS. Difficulties were, however, encountered with TVS imaging in the UKCTOCS. In a retrospective review of 1000 archived cases, ovaries and fallopian tubes could be identified in only 50% of cases^5^. TVS imaging could have been more reliable in the NROSS. Another difference between the trials relates to processing of blood for measurement of CA125. In the NROSS, blood was drawn in glass tubes without gel, serum was separated and frozen on the same day, while in the UKCTOCS blood was drawn in gel separation tubes, shipped at ambient temperature and separated after up to 56 h. A modest systematic reduction in CA125 levels in the UKCTOCS could have decreased the ability to detect early stage disease. In addition, particular care was taken in the NROSS to follow elevations of CA125 with repeated TVS and to minimize time to surgical intervention.

### Next steps in the early detection of ovarian cancer

The negative outcome of the UKCTOCS calls into question, where we should go next to find an effective strategy for early detection of ovarian cancer. While some might suspend attempts to detect early stage ovarian cancer awaiting a novel and disruptive technology, the two-step screening strategy has already achieved adequate specificity and a clear stage shift, although sensitivity is not yet adequate. There are opportunities for improvement both in serum biomarkers and in imaging. Only 80% of ovarian cancers express CA125 and serum levels of CA125 are elevated in only 70% of stage I/II cancers. A recent review identified 35 biomarkers that complement CA125 and could potentially improve sensitivity of the initial step in screening^6^. A combination of CA125, HE4, and CA72.4 detects 16% of cases missed by CA125^7^. Through a collaboration sponsored by the NCI Early Detection Research Network (EDRN), CA125 detected 72% of early stage cases at 98% specificity, whereas a combination of CA125, HE4 antigen-autoantibody complexes^8^ and osteopontin^9^ detected 89% at 94% specificity^10^. A second-generation ROCA algorithm is being developed and can be tested prospectively for specificity in the NROSS cohort.

In addition to detecting a greater fraction of early stage patients, panels of biomarkers could improve lead time with detection of cancers at longer intervals before clinical presentation. Autoantibodies could arise in response to very small volumes of early disease, which would be particularly important for high grade serous lesions arising from the fallopian tube. Anti-p53 autoantibodies have been detected in more than 20% of patients with early and late stage ovarian cancer^11^. Assaying serum samples from the UKCTOCS, titers of anti-p53 autoantibodies increased 8 months before elevation of CA125 and 22 months prior to clinical presentation in patients who did not exhibit increases in serum CA125^12^. This is the first of >120 biomarkers tested by our group that increased lead time over CA125. Among 19 promising autoantibodies tested, anti-p53, anti-CTAG1, and anti-IL-8 detected the greatest fraction of early stage ovarian cancer patients^11^.

A variety of additional biomarkers are being developed to detect ovarian cancer including ctDNA, methylated DNA, and miRNAs. Alterations in cervical and peripheral blood ctDNA can complement CA125 in detecting early stage disease^12^. While ovarian cancer has been included in DNA-based pan-cancer screening strategies^13,14^, detecting stage I/II disease has proven challenging. Future research should optimize the integration of DNA and protein biomarkers.

Whatever the biomarker panel chosen, screening could be performed more frequently. In patients at high risk, largely related to germ-line BRCA1/2 mutations, screening with the ROCA every 3 months proved more effective than annual screening^15,16^, While it is difficult to imagine more frequent screening for ovarian cancer alone in patients at conventional risk, blood might be drawn every six months to screen for multiple cancers in women over 50. Ovarian cancer screening could be paired with DNA-based pan-cancer screening strategies or combined with site-specific blood tests that are being developed to detect colorectal adenomas, and breast and pancreatic cancers^17^.

Imaging, the second step in two-stage screening, poses perhaps the greatest unmet need. As a single modality, TVS lacks adequate sensitivity and specificity for early detection of ovarian cancer. The majority of high-grade serous cancers probably arise in the fallopian tubes. Even in expert hands, fallopian tubes could not be imaged in 23% of 549 healthy women^18^. CT, PET-CT, and MRI also have problems with sensitivity, specificity, exposure to radiation and cost for screening^7^.

One possible solution in patients with rising serum biomarkers and negative TVS is falloposcopy, where a fiberoptic scope is threaded through the uterus and fallopian tube to visualize the fimbriae and ovary. This would be particularly relevant for women with BRCA1/2 mutations who are delaying risk reducing surgery. The EDRN is currently evaluating the feasibility of this approach. Another technology that is being developed is superconducting quantum interference detection (SQUID), which is a sensitive method for detecting faint magnetic fields. Anti-CA125 antibodies have been conjugated with ferritin nanospheres. Only antibody conjugated nanospheres bound to cells are detected by magnetic relaxation. Ex vivo, 10^6^ ovarian cancer cells (0.1 mm) can be detected^7^. If uptake of antibody-coated nanospheres can be optimized in xenografts, this approach might be utilized to detect recurrent ovarian cancer and then tested in healthy women with rising biomarkers and negative TVS.

## Conclusions

Given the specificity of the two-step screening strategy, opportunities to improve both phases and the impressive stage shift in the CA125-based NROSS trial, further development of this approach appears worthy of pursuit. The potential benefit for ovarian cancer patients is substantial. Computer simulations suggest that an effective strategy for early detection could reduce mortality by 10–30%, a dramatic improvement over our current attempts to improve therapy^7^.

## References

[1] Menon U (2021). Ovarian cancer population screening and mortality after long-term follow-up in the UK Collaborative Trial of Ovarian Cancer Screening (UKCTOCS): a randomised controlled trial. Lancet.

[2] Jacobs IJ (2016). Ovarian cancer population screening and mortality after long-term follow-up in the UK Collaborative Trial of Ovarian Cancer Screening (UKCTOCS): a randomised controlled trial. Lancet.

[3] Fan D (2021). A novel salt inducible kinase 2 inhibitor, ARN-3261, sensitizes ovarian cancer cell lines and xenografts to carboplatin. Cancers.

[4] Lu KH (2013). A 2-stage ovarian cancer screening strategy using the risk of ovarian cancer algorithm (ROCA) identifies early-stage incident cancers and demonstrates high positive predictive value. Cancer.

[5] Stott W (2018). Sonographers’ self‐reported visualization of normal postmenopausal ovaries on transvaginal ultrasound is not reliable: results of expert review of archived images in UKCTOCS. Ultrasound Obstet. Gynecol..

[6] Bast RC (2020). Biomarkers and strategies for early detection of ovarian cancer. Cancer Epidemiol. Biomark. Prev..

[7] Simmons AR (2019). Complementary longitudinal serum biomarkers to CA125 for early detection of ovarian cancer. Cancer Prev. Res..

[8] Yang WL (2019). Human epididymis protein 4 antigen-autoantibody complexes complement cancer antigen 125 for detecting early-stage ovarian cancer. Cancer.

[9] Guo J (2019). Osteopontin, macrophage migration inhibitory factor and anti-interleukin-8 autoantibodies complement CA125 for detection of early stage ovarian cancer. Cancers.

[10] Han, C. Y. et al. Multi-biomarker panel assessment of serological assays in early detection of ovarian cancer. In *12th EDRN Scientific Workshop* (2021).

[11] Yang WL (2017). Elevation of TP53 autoantibody before CA125 in preclinical invasive epithelial ovarian cancer. Clin. Cancer Res..

[12] Bast, R. C. Jr, et al. Critical questions in ovarian cancer research and treatment: report of an AACR Special Conference. *Cancer* **125**, 1963–1972 (2019) 10.1002/cncr.32004 PMC6557260 30835824

[13] Cohen JD (2018). Detection and localization of surgically resectable cancers with a multi-analyte blood test. Science.

[14] Lennon, A. M. et al. Feasibility of blood testing combined with PET-CT to screen for cancer and guide intervention. *Science*10.1126/science.abb9601 (2020). 10.1126/science.abb9601 PMC7509949 32345712

[15] Rosenthal AN (2017). Evidence of stage shift in women diagnosed with ovarian cancer during phase II of the United Kingdom Familial Ovarian Cancer Screening Study. J. Clin. Oncol..

[16] Skates SJ (2017). Early detection of ovarian cancer using the risk of ovarian cancer algorithm with frequent CA125 testing in women at increased familial risk—combined results from two screening trials. Clin. Cancer Res..

[17] Bast RC, Srivastava S (2020). The National Cancer Institute Early Detection Research Network: two decades of progress in cancer biomarkers. Cancer Epidemiol. Biomark. Prev..

[18] Lefringhaus JR (2016). Probability of fallopian tube and ovarian detection with transvaginal ultrasonography in normal women. Women’s Health.

